# Construction and validation of a glioblastoma prognostic model based on immune-related genes

**DOI:** 10.3389/fneur.2022.902402

**Published:** 2022-07-28

**Authors:** Kate Huang, Changjun Rao, Qun Li, Jianglong Lu, Zhangzhang Zhu, Chengde Wang, Ming Tu, Chaodong Shen, Shuizhi Zheng, Xiaofang Chen, Fangfang Lv

**Affiliations:** ^1^Department of Pathology, First Affiliated Hospital of Wenzhou Medical University, Wenzhou, China; ^2^Department of Neurosurgery, First Affiliated Hospital of Wenzhou Medical University, Wenzhou, China; ^3^Department of Pediatric Pulmonology, The Second Affiliated Hospital and Yuying Children's Hospital, Wenzhou Medical University, Wenzhou, China

**Keywords:** glioblastoma, immune-related genes, nomogram, prognostic model, TNC, mass spectrometry

## Abstract

**Background:**

Glioblastoma multiforme (GBM) is a common malignant brain tumor with high mortality. It is urgently necessary to develop a new treatment because traditional approaches have plateaued.

**Purpose:**

Here, we identified an immune-related gene (IRG)-based prognostic signature to comprehensively define the prognosis of GBM.

**Methods:**

Glioblastoma samples were selected from the Chinese Glioma Genome Atlas (CGGA). We retrieved IRGs from the ImmPort data resource. Univariate Cox regression and LASSO Cox regression analyses were used to develop our predictive model. In addition, we constructed a predictive nomogram integrating the independent predictive factors to determine the one-, two-, and 3-year overall survival (OS) probabilities of individuals with GBM. Additionally, the molecular and immune characteristics and benefits of ICI therapy were analyzed in subgroups defined based on our prognostic model. Finally, the proteins encoded by the selected genes were identified with liquid chromatography-tandem mass spectrometry and western blotting (WB).

**Results:**

Six IRGs were used to construct the predictive model. The GBM patients were categorized into a high-risk group and a low-risk group. High-risk group patients had worse survival than low-risk group patients, and stronger positive associations with multiple tumor-related pathways, such as angiogenesis and hypoxia pathways, were found in the high-risk group. The high-risk group also had a low IDH1 mutation rate, high PTEN mutation rate, low 1p19q co-deletion rate and low MGMT promoter methylation rate. In addition, patients in the high-risk group showed increased immune cell infiltration, more aggressive immune activity, higher expression of immune checkpoint genes, and less benefit from immunotherapy than those in the low-risk group. Finally, the expression levels of TNC and SSTR2 were confirmed to be significantly associated with patient prognosis by protein mass spectrometry and WB.

**Conclusion:**

Herein, a robust predictive model based on IRGs was developed to predict the OS of GBM patients and to aid future clinical research.

## Introduction

Glioblastoma multiforme (GBM) is the most common malignant brain tumor and it has high mortality and morbidity. In the USA, GBM accounts for 14.7%, 47.7%, and 56.6% of all primary brain tumors, malignant brain tumors, and gliomas, respectively (1, 2). At present, treating GBM entails maximal surgical resection and subsequent application of radiation therapy (RT) plus chemotherapy. Chemotherapeutic regimens most often include the alkylating agent temozolomide (TMZ) according to the Stupp protocol, which has been shown to positively impact long-term outcomes (3, 4). However, there are some challenges that need to be addressed, including how to achieve complete resection of tumors based on their location in core or inoperable sites in the brain, as well as on their infiltration into neighboring healthy brain tissues. Even with aggressive and comprehensive treatment, cancer relapse cannot be completely avoided. Patients with GBM have a dismal prognosis, with a 5.6% 5-year OS rate and a median OS time of 12–15 months (5, 6). Considering the dismal survival outcomes of individuals with GBM and the low effectiveness of the current treatment regimens, there is a pivotal need to identify novel treatment targets as well as alternative therapeutic approaches.

The major functions of the human immune system are to modulate organ homeostasis, offer protection against infectious pathogens, and remove damaged cells. Research evidence shows that adaptive and innate immunity play indispensable roles in the onset of cancer and contribute to cancer progression and treatment efficacy (7, 8). Over the last few decades, immunotherapy has become a revolutionary anticancer therapy. It has shown considerable benefits, such as enhancing survival, in numerous cancers, such as lung cancer, melanoma, and breast cancer (9, 10). Past research has suggested that the central nervous system has immune privilege due to the presence of the blood–brain barrier. However, in 2015, Louveau defined a new route for lymphatic outflow from the brain along different channels parallel to the dural venous sinuses. Therefore, most antigen-presenting cells that leave the brain likely reach the lymph nodes in the deep neck, where they can prime T and B lymphocytes. This suggests that immunogens in the brain can generate a powerful immune response.

**Figure 1:**
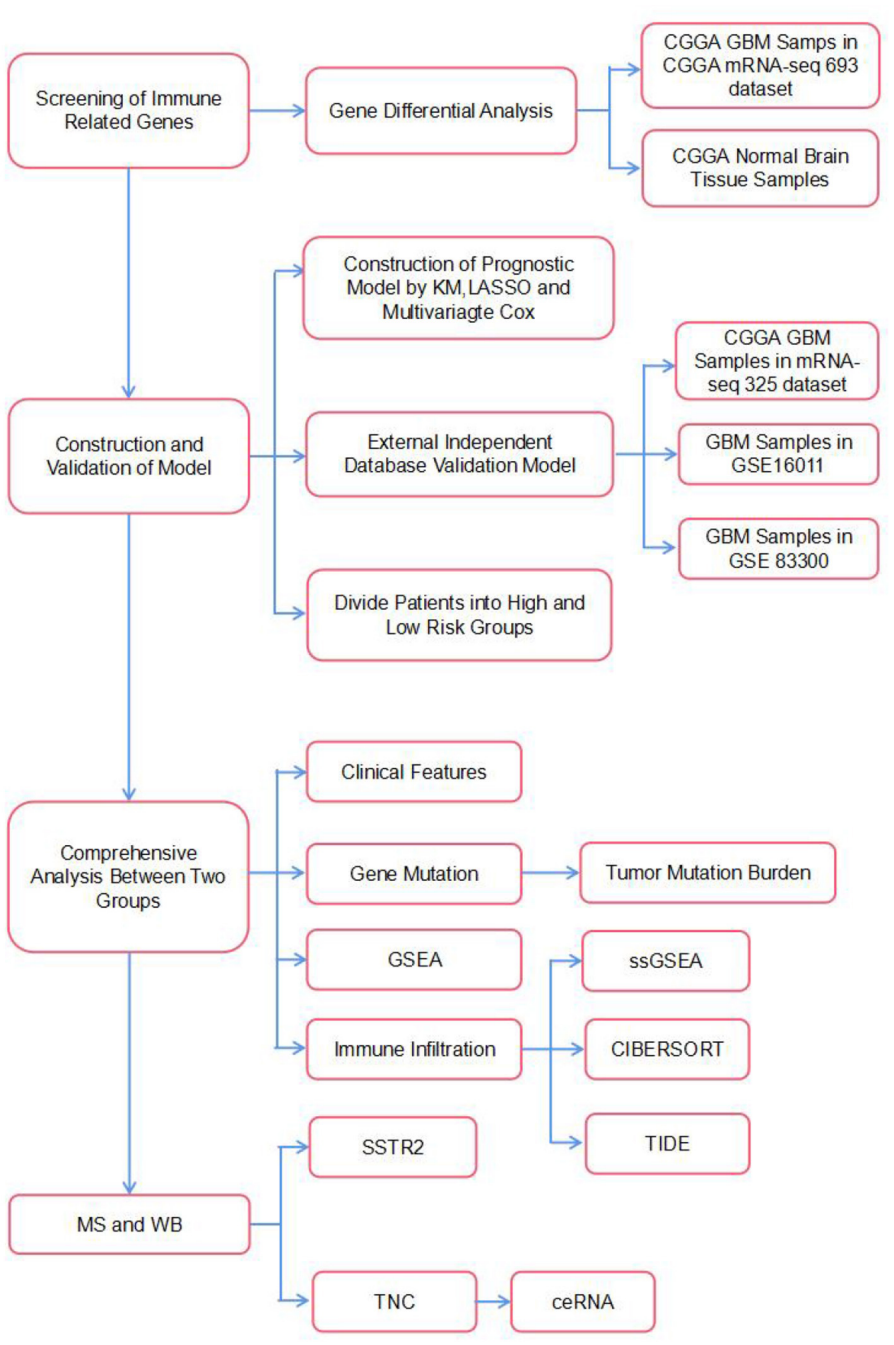
The flowchart of our study.

It is believed that although the brain is an immunologically unique site, the immune microenvironment provides ample opportunities to implement immunotherapy against brain tumors (11). Currently, numerous immunotherapeutic modalities for GBM have been proposed and established. They include immune checkpoint inhibitors, such as antibodies against cytotoxic T lymphocyte antigen 4 (CTLA-4), programmed cell death protein 1 (PD-1), or its ligand programmed death-ligand 1 (PD-L1), as well as CAR-T, vaccine and oncolytic virus therapies (12). Generally, a combination strategy involving immunotherapy, surgery, and chemoradiotherapy has been proposed as a prospective effective approach for treating GBM. Therefore, the purpose of the current study was to identify an IRG-based prognostic signature to comprehensively define the prognosis of GBM. In our study, six IRGs (CRH, CRLF1, SERPINA3, SSTR2, TNC, and TNFRSF19) closely associated with OS in GBM were identified using univariate and LASSO Cox regression analyses and used to construct a model to predict survival in GBM patients. We then characterized the molecular and immune features of our model and examined its prognostic power for patients treated with immunotherapy. Finally, we used mass spectrometry and WB to verify that the expression of the proteins encoded by these IRGs differed between patients with long and short survival times and constructed a ceRNA regulatory network. The flow chart of the study is shown in Figure 1.

## Materials and Methods

### Study population

RNA sequencing (RNA-seq) data and survival information of GBM patients in the CGGA mRNAseq_693 dataset were used as the training dataset. RNA sequencing data of normal brain tissue were obtained from the CGGA mRNA sequencing (using non-glioma as a control) dataset. RNA-seq data and survival information used for external model validation were obtained from the Gene Expression Omnibus (GEO, http://www.ncbi.nlm.nih.gov/geo) and CGGA databases. The GBM patients in the independent mRNAseq_325, GSE16011, and GSE83300 datasets were used as the validation datasets. MicroRNA expression data and lncRNA expression data were obtained from the microRNA_198 and mRNA-array_301 datasets in CGGA. Finally, gene mutation information was also obtained from the CGGA database.

### Differential expression analysis

The gene list comprising 1793 IRGs was obtained from the ImmPort data resource. The “limma” package in R was used for differential analysis, and the normalize between Arrays function in the “limma” package was used to normalize the gene expression profile (13). IRGs that were differentially expressed had *P* 0.05 along with absolute log2-fold change (FC) 1.5. GO functional enrichment and KEGG pathway enrichment analyses of differentially expressed IRGs were performed using the GO database (http://geneontology.org) and KEGG database (http://www.genome.jp/kegg/) (14, 15).

### Construction and validation of the prognostic model

Patients in the CGGA mRNAseq_693 dataset were used as a training cohort to construct our prognostic model. Univariate Cox regression and LASSO-Cox regression analyses were used to screen for IRGs that were significantly associated with survival (16). The risk score formula was calculated as follows: [−0.0475×expression value of corticotropin releasing hormone (CRH)]-[0.0260×expression value of cytokine receptor-like factor 1(CRLF1)]+[0.0640×expression value of serpin peptidase inhibitor, clade A (alpha-1 antiproteinase, antitrypsin), member 3(SERPINA3)]-[0.0162×expression value of somatostatin receptor 2(SSTR2)]+[0.0456×expression value of tenascin C(TNC)]+[0.0272×expression value of tumor necrosis factor receptor superfamily, member 19(TNFRSF19)]. Each sample's risk score was calculated by multiplying the expression values of the specific genes by their weights in the Cox model and then summing the products. Patients were clustered into high- and low-risk groups according to the median risk score. Kaplan–Meier analysis was used to compare the difference in survival between the two groups. Then, receiver operating characteristic (ROC) curves for 1–3-year survival were drawn. The area under the curve (AUC) was used to estimate the sensitivity and specificity of the model for survival prediction. Finally, we used three independent datasets (CGGAmRNAseq_325, GSE16011 and GSE83300) to validate our prognostic model (17–19).

### Construction of the nomogram

Stepwise multivariate Cox regression analysis was used to assess independent prognostic indicators, including the radiotherapy status, chemotherapy status, and risk score. Afterwards, these factors were used to construct a nomogram, which was adopted to predict 1–3-year OS of patients with GBM. The ROC curves, calibration curves and decision curve analysis (DCA) curves were compared to determine the predictive accuracy of the prognostic model (20).

### Comprehensive analysis of the risk score

Gene set enrichment analysis (GSEA) was performed using GSEA software (GSEA v4.1.0, http://www.broadinstitute.org/gsea). Our analysis was based on HALLMARK and KEGG gene sets. The package “ggplot2” was used to visualize the GSEA results. Then, the package “maftools” in R was used to visualize the somatic mutations in the GBM patients with genetic mutation data from the CGGA database. We also grouped the patients according to their different clinical characteristics (age, sex, IDH mutation, MGMT promoter methylation and 1p19q codeletion) and compared the differences in the risk scores among the clinical characteristics subgroups. The relationship between IRGs and gene functional status was analyzed by CancerSEA (http://biocc.hrbmu.edu.cn/CancerSEA/) (21).

### Immune characteristics analysis and immunotherapy analysis

Enrichment scores for 16 immune cells and 13 immune-related functions were estimated using single-sample gene set enrichment analysis (ssGSEA). Then, 22 immune cell types were quantified using the R package “CIBERSORT.” Only samples with a CIBERSORT output *p*-value < 0.05 were screened for this study. We also compared the expression levels of immune checkpoints between the high-risk and low-risk groups. Recent studies have revealed two distinct mechanisms of tumor immune evasion. In some tumors, although cytotoxic T cells are highly infiltrated, these T cells are often dysfunctional. In other tumors, immunosuppressive factors can eliminate T cells infiltrating tumor tissue. Peng Jiang et al. (22) designed a novel computational architecture, the Tumor Immune Dysfunction and Rejection (TIDE) score, to integrate these two tumor immune escape mechanisms. We explored the predictive power of our immunotherapy response prognostic model with the TIDE website (http://tide.dfci.harvard.edu). These immune-related characteristics were compared between the high- and low-risk groups (23).

### Liquid chromatography-tandem mass spectrometry

Ten GBM samples were selected, of which five were from patients with short survival times (OS <1 year) and five were from patients with long survival times (OS >3 years). Clinical information for 10 patients is presented in Supplementary Table 1. Samples were taken from storage at −80°C. Equal amounts of protein were taken from each sample for enzymatic hydrolysis, and the peptides were labeled according to the instructions of the TMT kit. Peptides were fractionated by high pH reverse-phase HPLC on an Agilent 300 Extend C18 column (5 μm particle size, 4.6 mm ID, 250 mm length). The peptides were dissolved in phase A of the liquid chromatography mobile phase and separated using an EASY-nLC 1,000 ultrahigh-performance liquid chromatography system. MS data were retrieved using MaxQuant 1.5.2.8. The quantitative method was set to TMT-10plex, and the FDR of protein identification and PSM identification were both set to 1%. *P*-values < 0.05 and absolute FDR values >2 were considered differentially expressed proteins.

### Western blotting

GBM tissues (*n* = 4) cryopreserved in liquid nitrogen after surgery at the First Affiliated Hospital of Wenzhou Medical University were collected. Clinical information for 4 patients is presented in Supplementary Table 2. Total proteins were extracted and quantified using a bicinchoninic acid (BCA) assay. The antibodies used were: anti-TNC (Abcam ab108930), anti-SSTR2 (Abcam ab229007), anti-SERPINA3 (Abcam ab205198), anti-TNFRSF19 (Abcam ab96220), and anti-GAPDH (Abcam ab8245). All primary antibodies were rabbit anti-human antibodies. The secondary antibody was a goat anti-rabbit antibody. Briefly, equal amounts (40 μg) of protein from each sample (tumors and control tissues) were separated by 10% SDS-PAGE electrophoresis and then transferred to PVDF membranes. The PVDF membranes were incubated with the primary antibodies, followed by the secondary antibodies, and then visualized. GAPDH was used as an internal reference for the western blot analysis.

### Construction of the ceRNA regulatory network

Co-expression analysis was used to screen miRNAs that regulated mRNAs and lncRNAs that competed with miRNAs for binding. A *p*-value < 0.05 and an *R-*value >0.3 were considered to indicate a significant correlation. Then, the mRNA targets of miRNAs and miRNA targets of lncRNAs were analyzed with the ENCORI web server (http://starbase.sysu.edu.cn/). RNAs with identical results in coexpression analysis and ENCORI analysis were suggested as possible components of a ceRNA regulatory network.

### Statistical analyses

Differences between the high-risk and low-risk groups were compared by the Wilcoxon test. Survival analysis was performed using the log-rank test. Multivariate survival analysis was performed using Cox regression analysis. Correlation analysis was performed using the Spearman method, and a two-sided *p* < 0.05 was considered significant. Statistical analysis was performed in R 4.0.3.

## Results

### Differentially expressed immune-related genes

**Figure 2:**
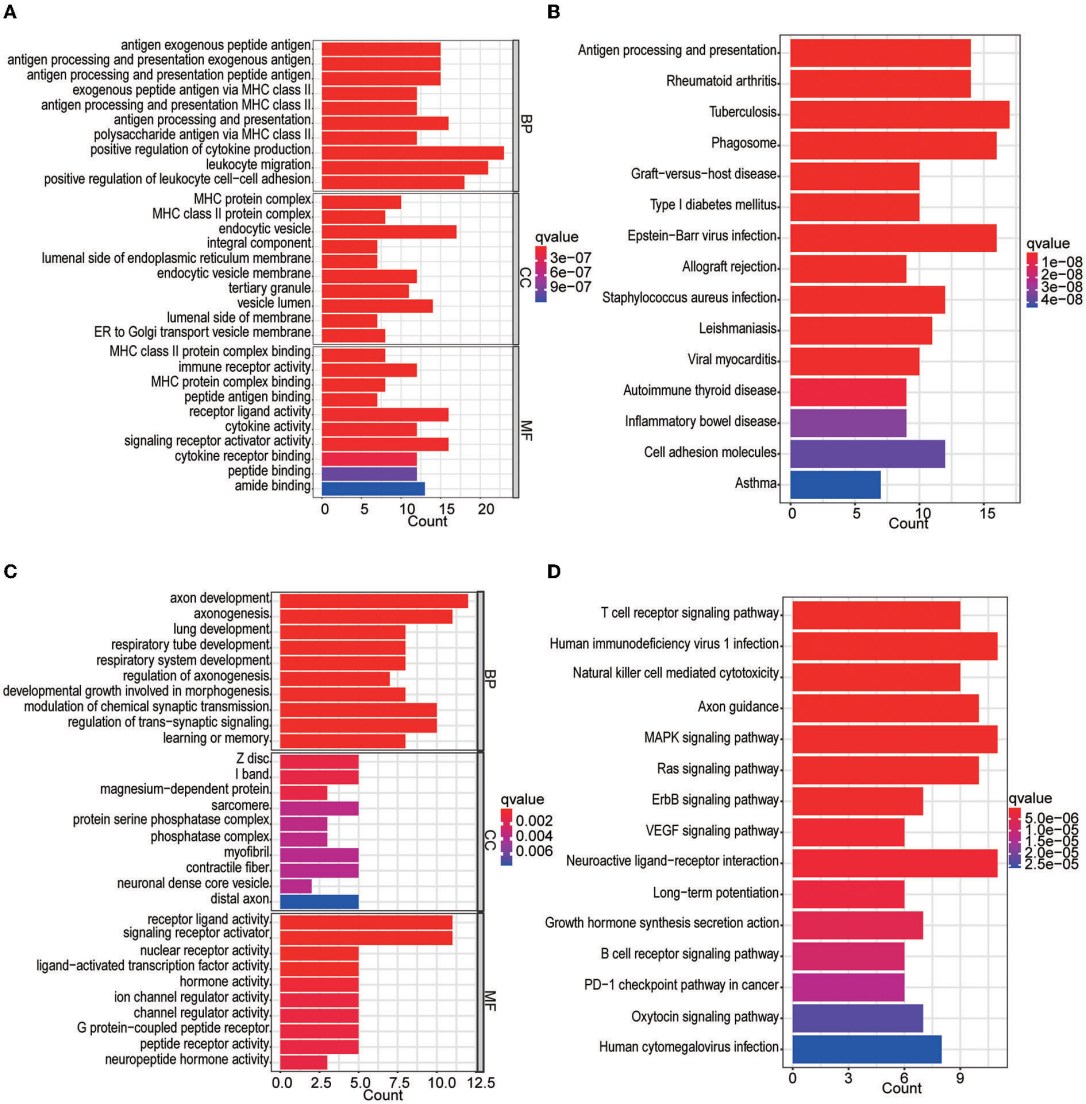
GO and KEGG enrichment analyses of differentially expressed IRGs. **(A)** GO analysis of upregulated IRGs. **(B)** KEGG analysis of upregulated IRGs. **(C)** GO analysis of downregulated IRGs. **(D)** KEGG analysis of downregulated IRGs.

In the differential expression analysis (249 tumor and 20 normal samples), a total of 142 differentially expressed IRGs were obtained; specifically, 89 IRGs were upregulated and 53 IRGs were downregulated in the tumor samples compared with the normal samples. The top 10 GOBP, GOCC, and GOMF terms and top 15 KEGG pathways are shown in Figure 2. We found that IRGs upregulated in tumor tissues were mainly enriched in activities and pathways related to antigen processing and presentation as well as MHC class II complexes. In contrast, IRGs downregulated in tumor tissues were mainly enriched in axon development, signaling receptor-related activities, and T-cell receptor signaling pathways.

### Glioblastoma prognostic signature

**Figure 3:**
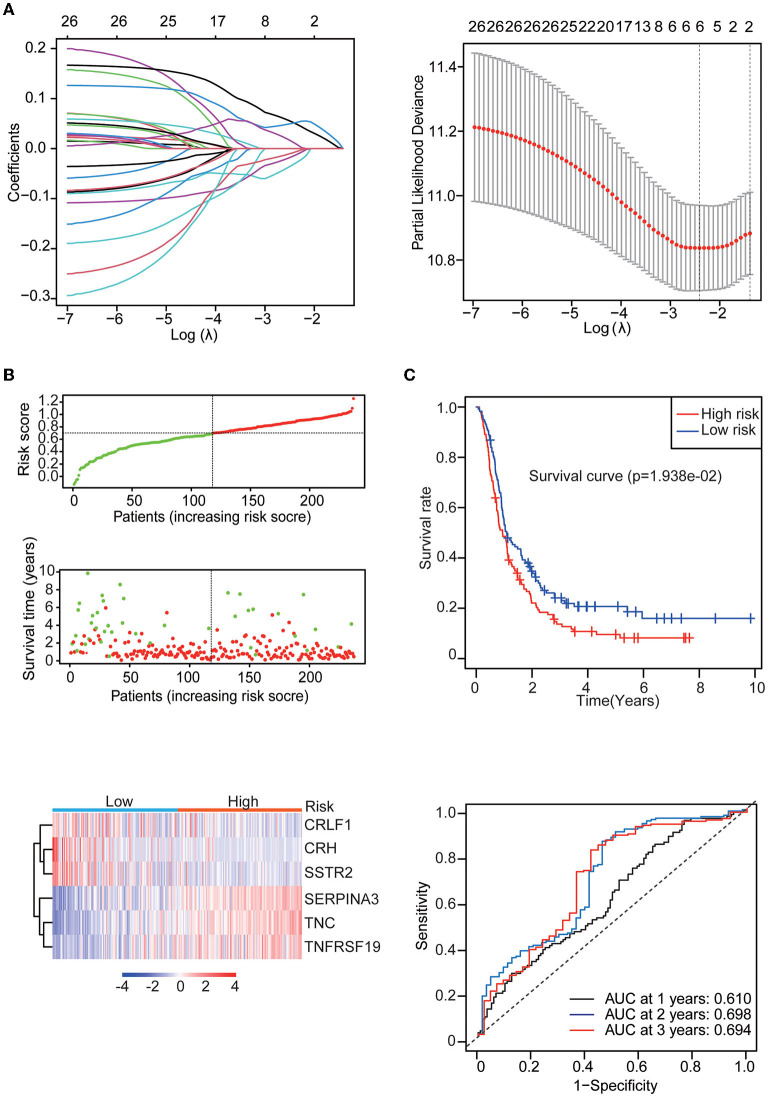
Identification of the IRG prognostic signature. **(A)** Coefficients of the determined characteristics are shown by the lambda parameter. The partial probability deviance relative to log (λ) was calculated *via* the LASSO Cox regression approach. **(B)** Prognostic assessment of the gene signature in the CGGA mRNAseq_693 cohort. Top: The dotted line designates the median risk score and stratifies the patients into low-risk GBM and high-risk GBM groups. Middle: Survival status of the patients. Bottom: Heatmap showing the expression patterns of the prognostic genes in the low-risk GBM and high-risk GBM groups. **(C)** Kaplan–Meier survival analysis of patients stratified by the gene signature. Time-dependent ROC analysis of the gene signature.

Univariate Cox regression analysis identified 27 genes among the 142 differentially expressed IRGs. Then, the multiple regression model was trained using the features selected by LASSO Cox regression analysis. Finally, six genes (CRH, CRLF1, SERPINA3, SSTR2, TNC, and TNFRSF19) were obtained. By calculating each patient's risk score using the same formula, the patients were divided into high- and low-risk groups based on the median risk score (Figure 3B). Kaplan–Meier analysis showed significant differences in OS between the high-risk group and the low-risk group in the training set (*p* = 1.938e-02). In addition, time-dependent ROC analysis showed that the risk score could efficiently estimate the 1, 2, and 3-year OS probabilities. The results for the calculation of the 1-year AUC (0.610), 2-year AUC (0.698), and 3-year AUC (0.694) are presented in Figure 3C.

**Figure 4:**
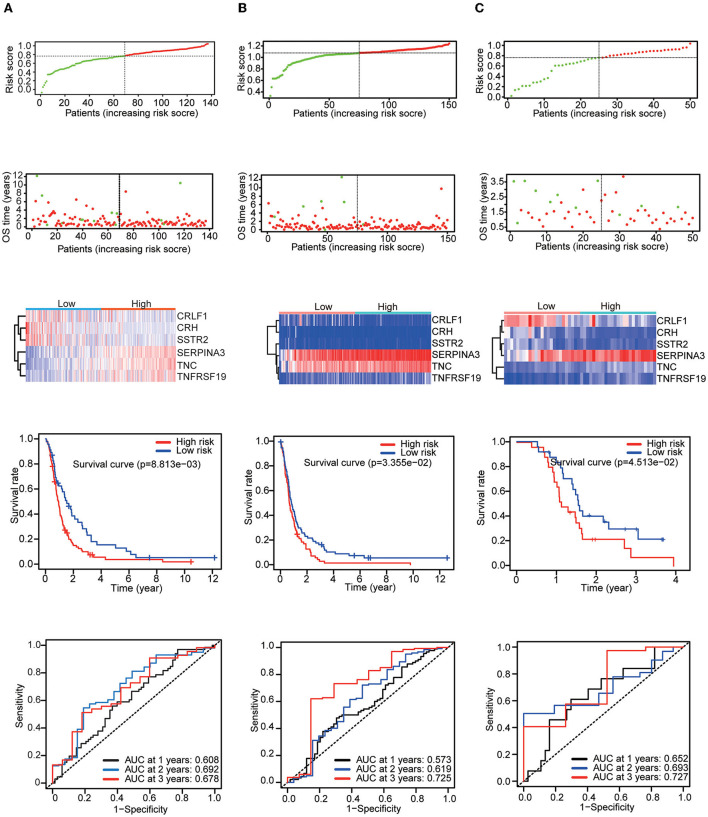
Patients in the validation set were used to verify the risk score model. Top: The dotted line designates the median risk score and stratifies the patients into low-risk GBM and high-risk GBM groups. Middle: Survival status of the patients. Heatmap of the prognostic genes in the low-risk GBM and high-risk GBM groups. Bottom: Kaplan–Meier survival curves and time-dependent ROC curves of the patients in the validation sets. **(A)** CGGA mRNAseq_325 dataset. **(B)** GSE16011 dataset. **(C)** GSE83300 dataset.

Then, three independent datasets – CGGA mRNAseq_325, GSE16011, and GSE83300 – were used to validate our prognostic model. The patients in each validation dataset were also divided into two groups based on their median risk score. There were also significant differences in the expression of six genes between the high-risk and low-risk groups. KM and time-dependent ROC analyses were performed in the three validation datasets. The results showed that our prognostic signatures were well-differentiated between the high- and low-risk groups. The prognostic model also accurately estimated the OS probability at 1–3 years (Figure 4).

### GSEA, gene mutation landscape, and clinical factor analysis

**Figure 5:**
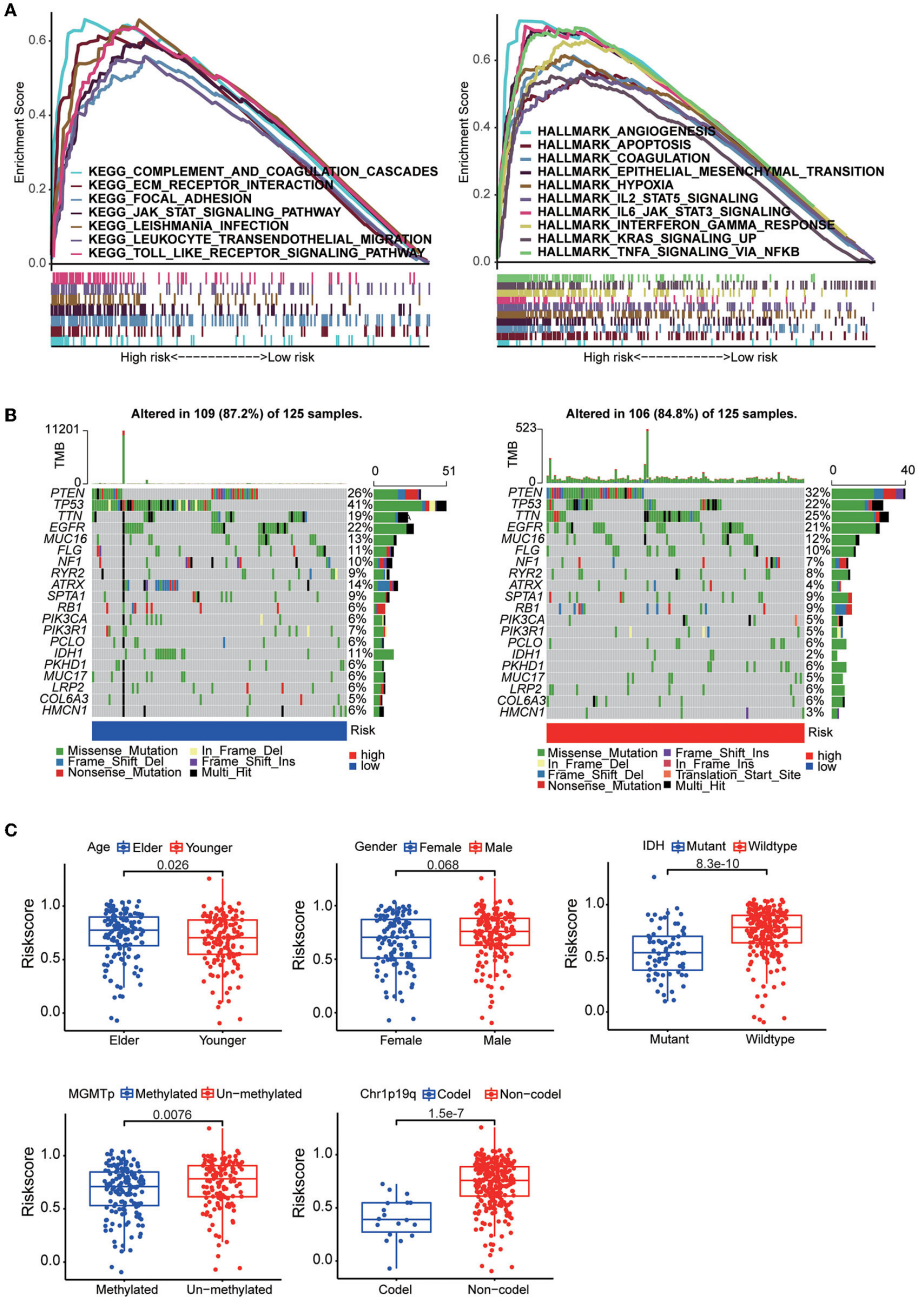
**(A)** GSEA between the high-risk group and the low-risk group based on the HALLMARK gene sets and KEGG gene sets. **(B)** The somatic landscape of the low-risk group samples and high-risk group samples. Mutation information for each gene in each sample is shown in waterfall plots, with different colors at the bottom representing specific annotations indicating various mutation types. **(C)** Boxplots showing the distribution of risk scores in GBM samples categorized by different factors, including age, sex, IDH mutation status, MGMT promoter methylation status, and chr1p19q codeletion status.

The results of KEGG analysis by GSEA showed that the complement system pathway, extracellular matrix (ECM) receptor pathway and cell adhesion pathway were enriched in the high-risk group. The results also showed that multiple hallmark gene sets associated with tumor development, including angiogenesis, hypoxia and epithelial-mesenchymal transition gene sets, were enriched in the high-risk group. Then, by mutation analysis, we found that IDH1 and TP53 gene mutations were more common in the low-risk group and PTEN mutations were more common in the high-risk group (Figure 5B). When the patients were grouped according to their clinical characteristics, a comparison of the differences in risk scores between the two groups demonstrated that younger patients had lower risk scores. In addition, patients with MGMT promoter methylation, IDH mutation, and 1p19q co-deletion had lower risk scores. The differences associated with all of these molecular features demonstrate a strong link between the risk score and the molecular tumor subtype (Figure 5C).

### Construction of the nomogram

**Figure 6:**
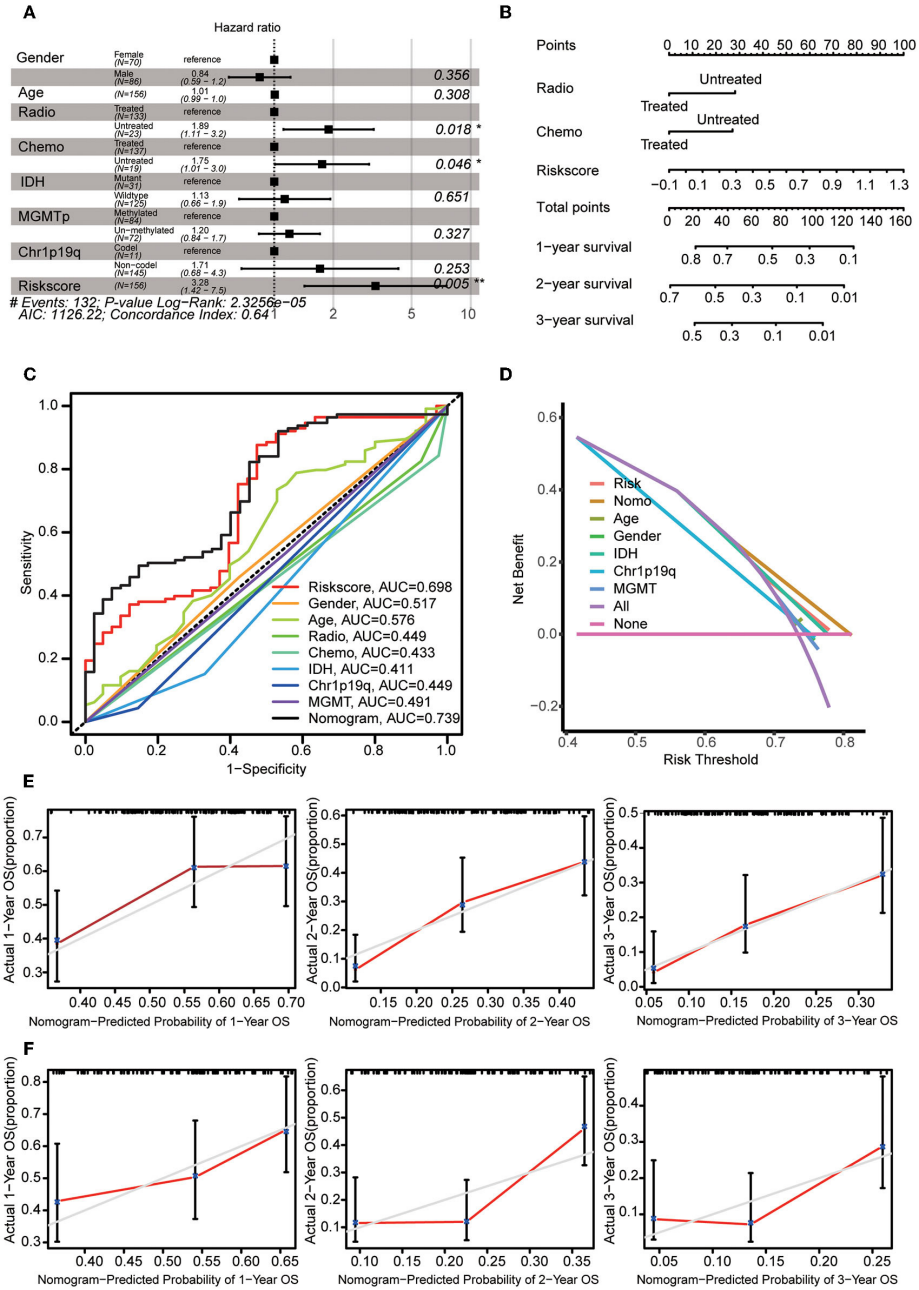
Construction of the nomogram. **(A)** Multivariate Cox regression analysis was adopted to select the independent variables, including radiotherapy status, chemotherapy status, and risk score. **(B)** The nomogram constructed using radiotherapy status, chemotherapy status and risk score. For each patient, three lines are drawn upwards to verify the points assigned from the three predictors of the nomogram. The sum of these points is located on the ‘Total Points' axis. Then, a line is drawn downwards to assess the 1–3-year overall survival probabilities of patients with GBM. **(C)** Multivariate ROC analysis was used to compare the predictive power of each variable. **(D)** DCA was used to compare the predictive power of each variable. **(E)** The calibration curve for the evaluation of the nomogram. The Y-axis shows the actual survival rate, while the X-axis shows the nomogram-estimated 1–3-year OS probabilities of patients in the training set. **(F)** The predicted 1–3-year OS probabilities of patients in the verification set.

Multivariate Cox regression analysis was used to explore the risk score as an independent predictor of survival. The data suggested that the risk score can be used as an independent variable to assess the prognosis of GBM patients (*p* = 0.005). In addition, radiotherapy and chemotherapy were also independent prognostic factors (Figure 6A). A nomogram was constructed to estimate the 1–3-year survival probabilities using the independent factors (radiotherapy status, chemotherapy status, and risk score) (Figure 6B). The multivariate ROC analysis showed that the nomogram had the largest AUC (Figure 6C), and the DCA results showed that the nomogram curve had the greatest deviation, both of which suggest that the nomogram has better predictive ability than any independent factor alone (Figure 6D). In the calibration curves for predicting 1–3-year survival, the red line indicates estimated survival, and the gray line indicates ideal survival. All three lines are closely aligned, showing good calibration in the CGGA mRNAseq_693 dataset (Figure 5E). Data from the validation set CGGA mRNAseq_325 were also acceptable in terms of predictive power (Figure 6F).

### Immune characteristics analysis in different risk groups

**Figure 7:**
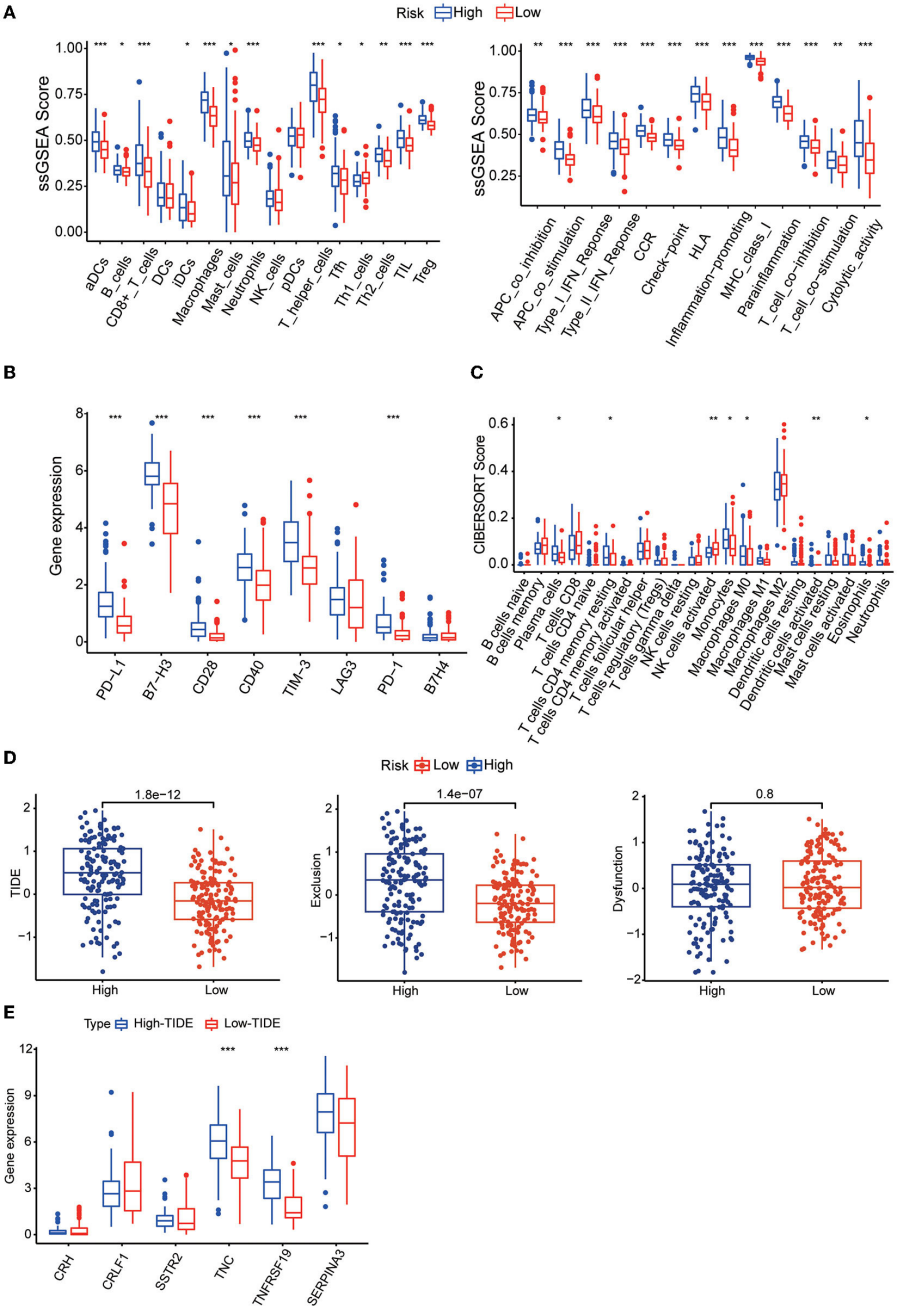
**(A)** Boxplots showing the levels of 16 immune cells in the two groups determined using ssGSEA. **(B)** Boxplots showing the levels of 13 proteins with immune-linked functions in the two groups determined by using ssGSEA. **(C)** Boxplots showing the expression of immune checkpoint genes in the high- and low-risk groups. **(D)** Boxplots showing the infiltration levels of 22 immune cell infiltrates in the two groups using CIBERSORT analysis. **(E)** TIDE, T-cell dysfunction and exclusion scores in the two groups. The variables were compared between the two groups with the Wilcoxon test. *p < 0.05, **p < 0.01, ***p < 0.001.

To understand the relationship between the risk score and the immune microenvironment, ssGSEA-based enrichment scores were calculated for 16 immune cells and 13 proteins with immune-related functions. There were significant differences in 13 immune cells between the high-risk and low-risk groups, with the high-risk group having higher levels of CD8 T cells and TILs. Likewise, the high-risk group exhibited higher levels of all 13 proteins with immune-related functions than the low-risk group (Figure 7A). We also found that the expression levels of the immune checkpoints PD-1, PD-L1, B7-H3, CD28, CD40, and TIM3 were significantly higher in the high-risk group (Figure 7B). We then found by CIBERSORT analysis that resting memory CD4 T cells, plasma cells, monocytes, activated dendritic cells, eosinophils and M0 macrophages were more abundant in the high-risk subgroup, while activated NK cells were more abundant in the low-risk group (Figure 7C). These results revealed significant differences in the level of immune cell infiltration and immune activity between the high-risk group and the low-risk group. We also found higher TIDE scores and immune exclusion in the high-risk group than in the low-risk group, suggesting that the low-risk group would benefit more from immune checkpoint inhibitor (ICI) therapy (Figure 7D). We found that TNC and TNFRSF19 were significantly overexpressed in the high TIDE group (Figure 7E).

### Validation of differential protein expression and construction of a ceRNA regulatory network

**Figure 8:**
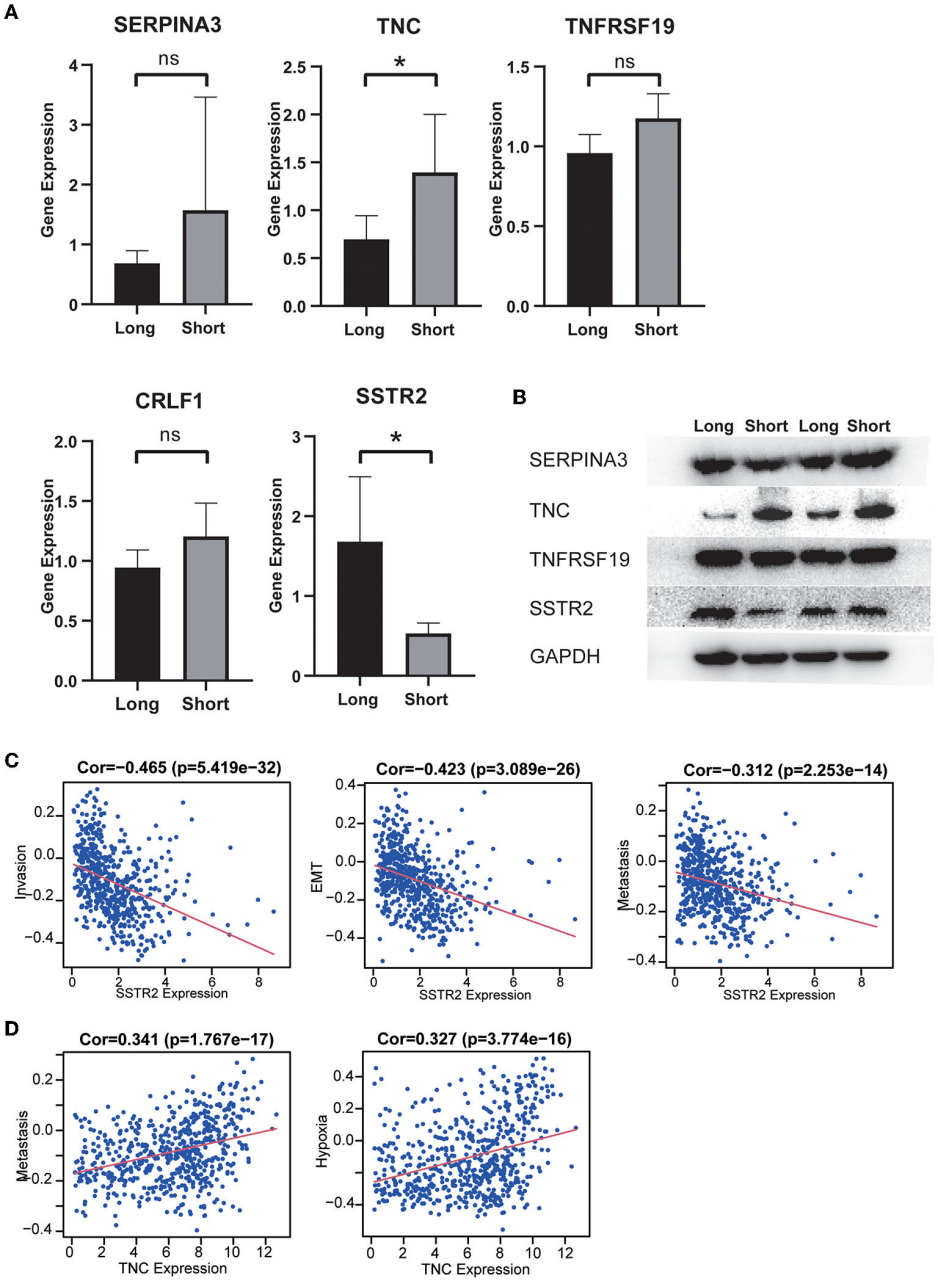
**(A)** The expression distribution of risk score IRGs in the short-survival time group and the long-survival time group. The asterisks represent levels of significance *p < 0.05, ns: not statistically significant. **(B)** Western blot analysis of four differentially expressed IRGs in the signature model. The protein expression level of TNC in the short survival group was significantly upregulated, and the protein expression level of SSTR2 was significantly downregulated. There was no significant difference in the expression of SERPINA3 and TNFRSF19 between the two groups. **(C)** High expression of SSTR2 was negatively related to tumor invasion, EMT and metastasis. **(D)** High expression of TNV was positively related to tumor metastasis and hypoxia.

Our MS results showed that long-term survivors had higher expression of SSTR2 and lower expression of TNC than short-term survivors, and these results were consistent with our prognostic model. The differences in the expression of CRLF1 and SERPINA3 were not statistically significant, and the expression of CRH could not be detected in the samples (Figure 8A). Our WB results are consistent with the MS results, with long-term survivors having higher expression of SSTR2 and lower expression of TNC than short-term survivors, with no statistically significant differences in SERPINA3 and TNFRSSF19 expression (Figure 8B).

**Figure 9:**
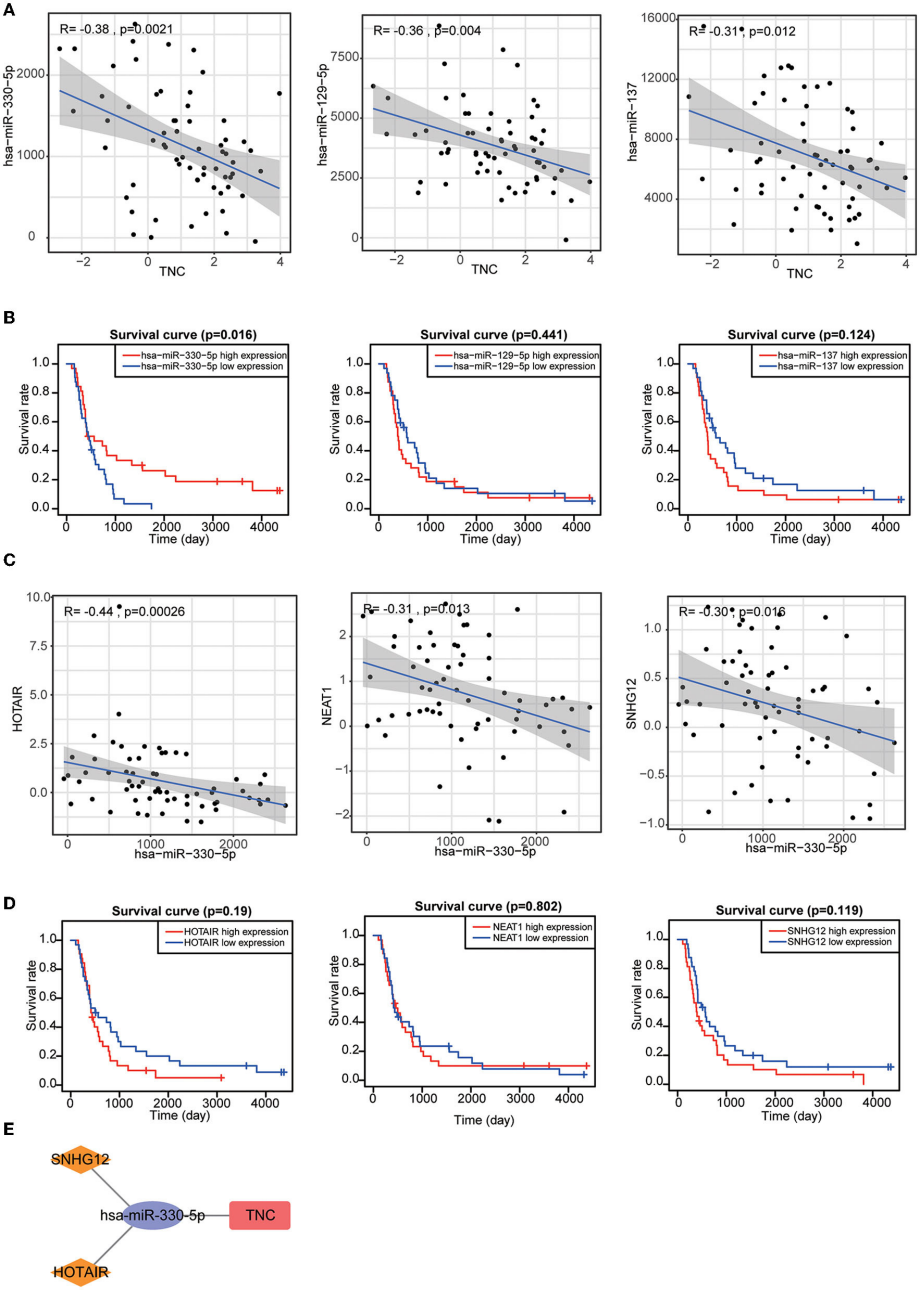
**(A)** Target miRNAs significantly negatively correlated with TNC. **(B)** KM curves of TNC-targeting miRNAs. **(C)** Target lncRNAs significantly negatively correlated with miR-330-5p. **(D)** KM curves of miR-330-5p-targeting lncRNAs. **(E)** HOTAIR and SNHG12 can regulate TNC expression by competitively binding to miR-330-5p.

The results of single-cell analysis with the CancerSEA database showed that high expression of SSTR2 was negatively related to invasion, EMT and metastasis. High expression of TNC was positively related to hypoxia and metastasis (Figures 8C,D). We attempted to construct separate ceRNA regulatory networks for TNC, SSTR2 and TNFRSF19. However, consistent results between co-expression analysis and ENCORI analysis were obtained only for TNC. We found that the expression levels of miR-330-5p, miR-129-5p, and miR-137 were significantly negatively correlated with that of TNC and that these miRNAs were TNC targets in ENCORI (Figure 9A). Because high expression of miR-330-5p was considered beneficial, we searched for targeted lncRNAs that were significantly negatively related to miR-330-5p (Figure 9B). HOTAIR, NEAT1, and SNHG12 were found to be significantly negatively related to miR-330-5p and HOTAIR, and SNHG12 had a strong correlation with a poor prognosis (Figures 9C,D). The lncRNA–miRNA–mRNA ceRNA regulatory network is shown in Figure 9E.

## Discussion

GBM, an aggressive primary malignant brain tumor, is common in adults. Currently, treatment strategies for GBM consist of surgery alone for early-stage disease and adjuvant radio/chemotherapy integrated with surgical resection for advanced-stage disease. However, the outcome of most GBM patients remains poor. For instance, surgical resection does not yield a satisfactory outcome since the GBM cells readily metastasize (24). In addition, there is still controversy as to whether systemic adjuvant treatment should be administered after surgery, considering the potential adverse effects and tumor heterogeneity (25).

In recent years, novel immunotherapies targeting the glioma immune microenvironment have shown great promise in the clinical management of tumors. Among various treatment strategies, drugs targeting immune checkpoint molecules have been hailed as breakthroughs (26–28). However, the efficacy of immunotherapy varies greatly and is affected by many factors. Sex, race, aging, obesity, exercise, alcohol consumption, and other factors have all been reported to affect the efficacy of immunotherapy. Compared with Asian Americans and Caucasians, people of African descent have a higher risk of certain malignancies. In addition, estrogen may promote higher levels of Treg cells, and female patients with malignant tumors are more likely to benefit from immunotherapy. For lifestyle habits, smoking and alcohol consumption can result in higher tumor mutational burdens and increased responsiveness to immunotherapy. A high-sugar, high-calorie diet reduces IL-17 levels and the benefit of immune checkpoint therapy. Finally, obese patients have high levels of TNF-α, IL-6, and IL-8 due to chronic inflammation, and when given immunotherapy, they live significantly longer (29–32). Therefore, it is essential to identify critical biomarkers to predict the outcomes of GBM patients. In the current study, an immune-related gene (IRG)-based prognostic signature was explored as a comprehensive approach to predicting the outcome of glioblastoma (GBM) and it exhibited significance in most analyses.

Many recent investigations have focused on the association of IRG expression with the onset and progression of diverse cancers (33). Comprehensive research evidence has documented that IRGs have a stable capacity to estimate the prognosis of patients, and numerous IRGs with robust estimation roles have been identified (34). To date, some existing nomograms have employed IRGs as predictive factors for individuals with glioma. A recent study established a nomogram with immune-linked gene pairs for estimating the survival of individuals with GBM (35), and a risk model based on 20 differentially expressed IRGs was demonstrated to exhibit efficient OS estimation potential in LGG (36).

However, the models established in past studies often do not have high AUC values and are not externally validated using independent datasets. Furthermore, they did not perform experimental validation of the genes included in the model. In our study, after a series of analyses based on the CGGA dataset, a prognostic signature consisting of six IRGs (CRH, CRLF1, SERPINA3, SSTR2, TNC, and TNFRSF19) was identified, and a nomogram with good predictive power was constructed, combining the risk score, radiotherapy status and chemotherapy status. Of the two IRGs validated by protein mass spectrometry and WB, TNC participates in vasculogenic mimicry (VM) formation, which is the generation of vessel-like structures by highly infiltrative tumor cells. VM has been regarded as one of the numerous mechanisms that account for the failure of antiangiogenic treatment in individuals with glioma (37). It has also been reported that TNC is a promoter of the invasiveness of brain tumor-initiating cells through a mechanism involving ADAM-9 proteolysis *via* the c-Jun NH2-terminal kinase pathway (38). SSTR2 was reported to be associated with favorable outcomes in various solid tumors. Appay et al. (39) validated that high expression of the SSTR2A protein was associated with a lower proliferation index, the absence of microvascular proliferation and the absence of necrosis, leading to better overall survival and progression-free survival.

We found that the risk score was associated with multiple tumor development-related pathways. Patients in the high-risk group were older and had lower IDH1 mutation rates, lower 1p19q co-deletion rates and lower MGMT promoter methylation rates. All of these differences would also lead to poor survival. Then, ssGSEA and CIBERSORT analysis showed that the infiltration level of NK cells in the high-risk group was significantly lower than that in the low-risk group, and the expression of MHC class I molecules was significantly higher in the high-risk group than in the low-risk group. We know that glioma cells can inhibit antigen-presenting cell-mediated recognition and NK-cell-mediated killing by expressing MHC class I molecules that interact with NK-cell immunoglobulin-like receptors.

We also found higher expression levels of PD-1 and PD-L1 in the high-risk group than in the low-risk group. PD-1 negatively regulates T-cell receptor-mediated signal transduction pathways, binds to PD-L1, inhibits its activation and cytotoxic T-cell effects, blocks the production of inflammatory factors, and leads to T-cell inactivation. Tumor-expressed PD-L1 is regulated by multiple mechanisms, including activation of the phosphatidylinositol 3-kinase (PI3K) signaling pathway and TIL-secreted interferon gamma (IFN-γ). In gliomas, PD-L1 is mainly expressed on tumor cells and TAMs and is negatively correlated with patient prognosis (40–42). In addition, patients in the high-risk group had higher TIDE scores and exclusion scores, demonstrating worse immunotherapy outcomes.

The recently proposed concept of ceRNAs plays an important role in the development of cancer. We constructed a TNC-centered ceRNA regulatory network, which helped us to explain the regulatory mechanism by which high TNC expression leads to a poor prognosis of GBM patients. It has important guiding significance for future research. In summary, our study utilized public databases to construct a prognostic model of six IRGs and validated it at the protein expression level. HOTAIR and the SNHG12–miR-330-5p–TNC axis might promote tumor progression *via* tumor cell metastasis and tumor hypoxia.

### Limitations

There are some limitations of this research. First, although we used MS/MS and WB to validate the protein expression differences of IRGs in the predicted model in GBM tissues with different prognoses, the small sample size may have biased the results. Second, *in vivo* and *in vitro* functional experiments are required to validate the functional importance of TNC and SSTR2. In addition, we assessed patients' immunotherapy susceptibility only with the TIDE score. We also need to detect the expression levels of IRGs in the prognostic model in a cohort of patients receiving immunotherapy, which will more intuitively reflect the predictive ability of our model for the response to immunotherapy.

## Conclusion

In this study, the immunogenomic landscape was analyzed, and an IRG-related prognostic signature for GBM was developed. The results of this study provide a more comprehensive understanding of the immune response in the TME and prospective immunotherapy targets for clinical use.

## Data Availability Statement

The original contributions presented in the study are included in the article/Supplementary material, further inquiries can be directed to the corresponding authors.

## Ethics Statement

The studies involving human participants were reviewed and approved by the Clinical Research Ethics Committee of the First Affiliated Hospital of Wenzhou Medical University. The patients/participants provided their written informed consent to participate in this study.

## Author contributions

Conception and design: KH and CR. Administrative support: QL and XC. Provision of study materials or patients: JL, ZZ, and CW. Collection and assembly of data: CS and SZ. Western Blot: FL. Data analysis and interpretation: FL and CR. Manuscript writing and final approval of manuscript: All authors.

## Funding

This research was supported by grants from Wenzhou Science and Technology Project under Grant, Y20190144.

## Conflict of interest

The authors declare that the research was conducted in the absence of any commercial or financial relationships that could be construed as a potential conflict of interest.

## Publisher's note

All claims expressed in this article are solely those of the authors and do not necessarily represent those of their affiliated organizations, or those of the publisher, the editors and the reviewers. Any product that may be evaluated in this article, or claim that may be made by its manufacturer, is not guaranteed or endorsed by the publisher.

## Supplementary Material

Table S1 Clinical data of 10 patients by liquid chromatography-tandem mass spectrometry.

Table S2 Clinical data of 4 patients in Western Blot.
