# Supplementary material for: Construction and validation of a glioblastoma prognostic model based on immune-related genes
Source: Front Neurol. 2022 Jul 28;13:902402. doi: 10.3389/fneur.2022.902402 (PMC9366078; doi:10.3389/fneur.2022.902402)
Supplement: Table S1 — Clinical data of 10 patients by liquid chromatography-tandem mass spectrometry. [file Data_Sheet_1.docx]

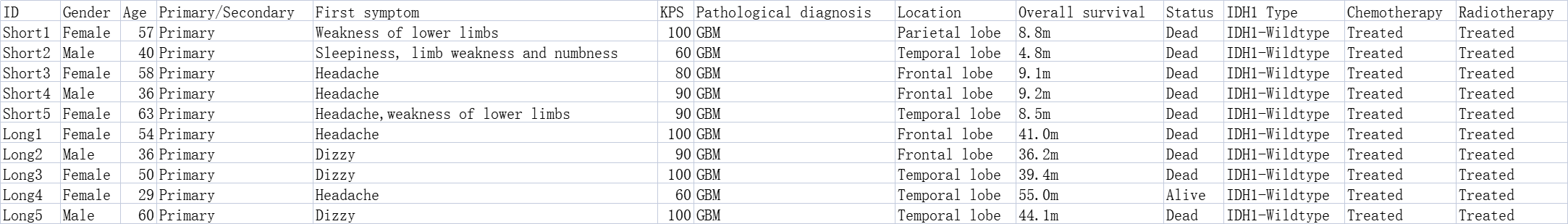


Supplementary Table 1:Clinical data of 10 patients by liquid chromatography-tandem mass spectrometry.


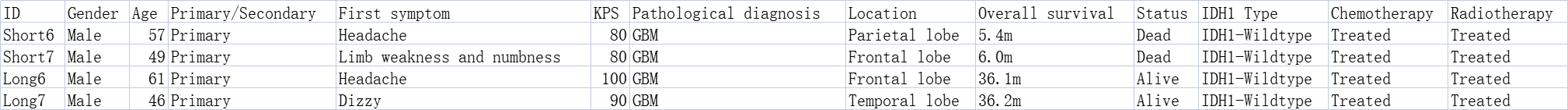


Supplementary Table 2:Clinical data of 4 patients in Western Blot.
